# Effect of growth hormone replacement therapy in a boy with Dent's disease: a case report

**DOI:** 10.1186/1752-1947-5-400

**Published:** 2011-08-22

**Authors:** Mira Samardzic, Snezana Pavicevic, Michael Ludwig, Radovan Bogdanovic

**Affiliations:** 1 Institute for Sick Children, Department of Endocrinology and Nephrology, Ljubljanska bb, 20 000 Podgorica, Montenegro; 2 Department of Clinical Chemistry and Clinical Pharmacology, University of Bonn, Bonn, Germany; 3 Institute for Mother and Child Health Care of Serbia, Department of Nephrology, Radoja Dakica 10, 11 000 Belgrade, Serbia

## Abstract

**Introduction:**

Dent's disease is an X-linked recessive proximal tubulopathy characterized by low molecular weight proteinuria, hypercalciuria, nephrocalcinosis, nephrolithiasis and progressive renal failure. To the best of our knowledge, this is only the third report on the use of growth hormone therapy in a child with poor growth associated with Dent's disease.

**Case presentation:**

We report on a 7-year-old Montenegrin boy with proteinuria, hypercalciuria, nephrocalcinosis, rickets and short stature with unimpaired growth hormone secretion. A molecular genetic analysis showed S244L substitution on the CLCN5 gene. After two years of conventional treatment with hydrochlorothiazide, laboratory tests revealed more prominent proteinuria, mild hypophosphatemia, increased values of alkaline phosphatase and features of rickets. Phosphate salts, calcitriol, potassium citrate and growth hormone were included in the therapy. After three years of therapy, his adjusted parental stature was 1.53 standard deviations higher than at the initiation of growth hormone therapy. His global kidney functions and levels of proteinuria and calciuria remained relatively stable. In spite of the growth hormone therapy, his tubular reabsorption of phosphate deteriorated.

**Conclusion:**

Treatment with recombinant human growth hormone may have a positive effect on final height in poorly growing children with Dent's disease and hypophosphatemic rickets. However, it is not possible to reach definite conclusions due to the small sample within the literature and the brief duration of the therapy.

## Introduction

Dent's disease is an X-linked recessive proximal tubulopathy characterized by low molecular weight proteinuria, hypercalciuria, nephrocalcinosis, nephrolithiasis, and slowly progressive renal failure in affected males. Renal acidification abnormalities are only rarely seen in Dent's disease, whereas the hypokalemic metabolic alkalosis associated with hyperreninemic hyperaldosteronism (Bartter-like syndrome) has been reported in a few patients [1]. Clinical characteristics of Dent's disease include familial tubular syndromes such as X-linked recessive nephrolithiasis, X-linked recessive hypophosphatemic rickets and low-molecular weight proteinuria with hypercalciuria and nephrocalcinosis in Japanese children [2]. Dent's disease is caused by mutations in the CLCN5 gene, which is located on the short arm of the × chromosome (Xp11.22). The CLCN5 gene encodes for the 746 amino-acid CLC-5 chloride channel that belongs to the voltage-gated chloride channel family (CLC-7, CLC-Ka and CLC-Kb) implicated in membrane excitability, transepithelial transport and possibly the regulation of cell volume [3]. The mechanism by which CLC-5 dysfunction results in hypercalciuria and the other features of Dent's disease remains unclear.

The different clinical features of Dent's disease makes treatment complex, and it requires simultaneous monitoring of the effects of therapy. There is still no clear strategy for the management of patients with this condition. Thiazide diuretics have been used to reduce urinary calcium excretion, and to prevent the recurrence of nephrolithiasis [4]. A high citrate diet preserved the renal function and delayed the progression of renal disease in CLC-5 knockout mice [5]. Rickets was a prominent feature in about one third of patients reported with Dent's disease. The recommended treatment is based on oral phosphate salts and calcitriol [6].

## Case presentation

A 7-year-old Montenegrin boy was initially referred to the pediatric endocrinology ward because of short stature. His height was 2.93 standard deviations (SD) below the mean. His mid-parental height was 181.6 cm (+1 SD). Our patient was born at term following an uneventful pregnancy with a birth weight of 3.95 kg and a birth length of 57 cm. His family history was negative for short stature, delayed puberty and renal disease. Except for dental caries, high palate and slight genus valgus, he had no other abnormalities. His blood pressure was normal and his bone age was 5 years. A laboratory test was positive for proteinuria. He had an elevated urinary calcium level and β2-microglobulin excretion. A renal ultrasound showed early medullary nephrocalcinosis. His levels of serum calcium, phosphorus and alkaline phosphatase were normal. His tubular reabsorption of phosphate (TRP) was decreased. His creatinine clearance test [7] was normal (92.8 ml/min/1.73 m^2^). No other electrolyte or metabolic abnormalities were observed. His overnight growth hormone (GH) profiles were normal (> 3 peaks of > 10 μg/L). An audiometry test was also unremarkable. In order to confirm a diagnosis of Dent's disease, molecular genetic analysis was performed one year later and showed a mutation in the CLCN5 gene, leading to S244L amino acid substitution. The mutation carrier, the patient's mother, was asymptomatic with slight hypercalciuria.

**Figure 1:**
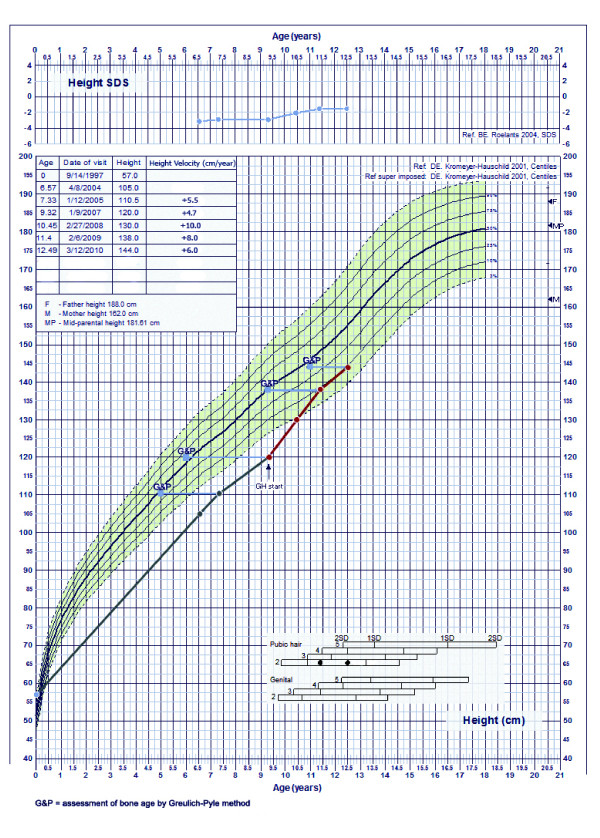
**Growth chart of child with Dent's disease before and during additional therapy with rhGH**. G&P: assessment of bone age by Greulich-Pyle method.

After two years of conventional treatment with hydrochlorothiazide, our patient was referred for a new endocrine evaluation because of a failure to catch-up growth. Laboratory tests showed more prominent urinary protein excretion, whereas the level of calciuria remained unchanged. Clinical, radiological and laboratory signs of hypophosphatemic rickets became noticeable. We found hypophosphatemia (0.74 mmol/L), elevated serum alkaline phosphatase activity (926 U/L), and a nearly normal level of parathyroid hormone. X-rays showed enlargement of his wrists and knees and fraying of the metaphyses of his distal ulna and radius. Our patient's growth velocity was 4.7 cm/yr (-1 SD). His parental adjusted height at that time was -3.11 SD; his pubertal status was Tanner stage one. A height prediction based on his recent growth was approximately 160.7 cm (Statistical program: SAS v9.13). At the age of nine years and three months we initiated recombinant human growth hormone (rhGH) therapy. The indication for GH therapy was markedly short stature and chronic renal disease stage one [8]. The average dose of rhGH was 0.04-0.05 mg/kg per day. Because of the overt hypophosphatemic rickets and hypercalciuria, in addition to hydrochlorothiazide we started him on calcitriol 20-40 ng/kg/day in two divided doses, phosphorus 20-40 mg/kg/day, maximum 2.5 g/day in 3-5 divided doses and potassium citrate. A follow-up was performed at three-month intervals. We followed his growth velocity, serum phosphate, serum creatinine, creatinine clearance, TRP, protein and calcium excretion, insulin-like growth factor 1(IGF-1), insulin-like growth factor-binding protein 3 (IGFBP-3) and other laboratory tests in relation to growth hormone therapy (Table 1, Table 2). In the first two years, our patient grew at an average rate of 9 cm per year (> 95c), and in the third year he grew 6 cm (50c) (Figure 1). His bone age remained retarded. During rhGH treatment and other therapies, there were no relevant changes in his creatinine clearance or the degree of nephrocalcinosis on renal ultrasonograms. His cystatin C level was also normal at -0.85 mg/L (normal range 0.53-0.95 mg/L). His level of proteinuria remained stable whilst urinary calcium excretion was reduced. Despite continued phosphaturia, his serum phosphate level increased gradually, and his serum alkaline phosphatase returned to normal. No acceleration in bone age or increase in glucose intolerance was noted. After suspending GH therapy for two months, GH secretion was re-evaluated: IGF-1 level was under the normal range and a clonidine stimulation test showed a peak serum GH concentration of 16.5 μg/l, again confirming the absence of a GH deficiency.

**Table 1:** Laboratory investigation before and after three years of combined conventional and GH replacement therapy

	24mo pre-GH	Baseline	24 mo post-GH	36mo post-GH
	**Hydrochlorothiazide**	**Hydrochlorothiazide + phosphate** **+ K-citrate** **+calcitriol+rhGH**	**Hydrochlorothiazide + phosphate** **+K-citrate +calcitriol+rhGH**	**Hydrochlorothiazide + phosphate** **+K-citrate** **+calcitriol+rhGH**

> 2 GH peaks at night (nl > 20 mU/L)	22.0-28.0	nd	nd	nd

GH peaks with provocative stimuli (nl > 20 mU/L)	nd	nd	nd	33 (2 mo without GH therapy)

IGF-1 (9 y: nl 123-275 ng/ml) (11 y: 139-395 ng/ml) (12 y: 143-693 ng/ml)	nd	122 (before GH therapy)	300.5 - 496 (with GH therapy)	90-125 (2 mo without GH therapy)

Calcium (s) (nl 2.1-2.5 mmol/L)	2.4	2.4	2.4	2.3

Phosphate (s) (nl 0.8-1.5 mmol/L)	1.0	0.74	0.96	1-1.25

iPTH (nl 0.95-5.7 pmol/l)	n.d	6.1	n.d	2.68

ALP (5-10 yr: 110-341 U/L) (Puberty: < 500)	224.70	926.0	638	160-174

TRP (nl 85-98%)	65-77	75	57	50-60

Creatinine clearance (nl 89-165 ml/min/1.73 m^2^)	92.8	99.8	107	112.7

β2-microglobulin (nl < 0.03-0.37 mg/24 h)	81.6	nd	nd	nd

Protein excretion (nl < 0.150 g/24 h)	1.86	3.21	2.1	2.0-3.0

Calcium excretion (nl < 4 mg/kg/24 h)	10.1	10.9	8.9	6.7-7.2

ALP: alkaline phosphatase; iPTH intact parathyroid hormone; nd: not determined; nl: normal level

**Table 2:** Anthropometric characteristics of rhGH-treated child with Dent's disease

	24mo pre-GH	Baseline	24 mo post-GH	36 mo post-GH
Age (y)	7.3	9. 3	11. 3	12. 3

Bone age (y)	4 4/12	6	9	11

Height (cm)	110.5	120.0	138	143.5

Height (SDS)	-2.89	-2.93	-1.83	-1.4

Sitting height (cm)	59	64	74	78

Sitting height/Leg length	1.16	1.14	1.19	1.18

BMI (SDS)	+1,26	+1.02	+1.16	+1.14

Parental adjusted height (SDS)	-3.08	-3.11	-2.02	-1.58

BMI: body mass index; SDS: standard deviation score

## Discussion

Dent's disease is an inherited tubulopathy caused by CLCN5 gene mutations. To date, more than 80 distinct CLCN5 mutations have been reported [9]. S244, is the most common mutation in CLCN5 thus far described. In our patient's family we also identified this mutation. Tosetto *et al. *found that approximately 48% of patients with Dent's disease had rickets, which correlates with only one mutation, S244L [10]. It is not completely clear how the loss of function in the endosomal chloride channel leads to a decrease in brush-border sodium/inorganic phosphate co-transport. However, not all patients with Dent's disease show a decrease in phosphate reabsorption [9]. Hoopes *et al*., found that hypophosphatemic patients were not always affected by rickets. Also, some patients with Dent's disease have been observed to have extrarenal manifestations such as mild intellectual impairment, hypotonia and cataracts, and such patients have been reported to share a mutation in OCRL1 with the oculocerebrorenal syndrome of Lowe. The occurrence of these extrarenal manifestations with mutations relating to Lowe syndrome is referred as Dent's disease type 2 [11].

The most striking physical sign in the first described patient with Dent's disease and hypophosphatemic rickets was the shortness of stature [12]. At the time of setting the differential diagnosis our patient also had growth failure (-2.89 SD), although he had normal global kidney functions. Sheffer-Babila *et al. *[13] studied the case of two brothers, 10 and 13 and a half years old, suffering from Dent's disease and GH deficiency, without symptoms of rickets. At the time of setting the diagnosis their growth retardation was -2.2 and -1.2 SD respectively. One brother had a diminished estimated glomerular filtration rate (GFR) (creatinine clearance: 68-83 ml/min/1.73 m^2^); the other had normal estimated GFR (creatinine clearance: 101-143 ml/min/1.73 m^2^). These patients were treated with enalapril, hydrochlorothiazide, calcitriol, phosphate supplements, vitamin E, vitamin C, potassium citrate and growth hormone. Two years after initiating GH therapy their growth velocity was 8 and 10 cm/yr respectively. In cases of short stature of various origins but without GH deficiency, such as Turner syndrome or short children born small for gestational age (SGA), the treatment used is rhGH. Not all patients with Dent's disease have GH deficiency, but reasons for treatment in our case were the presence of short stature and chronic renal disease [8]. In the first two years after starting our patient on GH and other therapies, the boy grew 9 cm/yr, and in the third year he grew 5.5 cm. His IGF-1 levels were below normal range before treatment and increased to normal levels after treatment. The acceleration in growth velocity could be attributed to the increased concentration of circulating IGF-1, the increase in efficiency of food utilization with rhGH, and the conventional therapy for hypophosphatemic rickets. Pharmacologic treatment of X-linked hypophosphatemia rickets leads to an improvement in the rickets, but effects on longitudinal growth and renal phosphate reabsorption are often disappointing [14].

Unlike in the case of the two brothers reported by Shaffer-Babila *et al.*[13], we did not observe an effect on renal phosphate reabsorption by rhGH treatment. It is well known that GH, at least where mediated by IGF-1 (locally produced in the kidney), stimulates proximal tubular sodium/inorganic phosphate co-transport [15].

The growth of our patient was improved and proportional to his pubertal state. Since the therapy was started before the patient reached puberty it is not possible to estimate how the GH therapy will continue to affect our patient's final height. However after two years of therapy, growth started to slow down.

Although a correlation was found between renal function and growth impairment, significant short stature was seen at all levels of renal function. The etiology of growth delay in children with chronic kidney disease is multifactorial, including rickets, GH resistance, reduced GH secretion rate or greater loss of GH, functional IGF deficiency and increased IGFBP -1,-2,-4 and -6 [16].

The aim of therapy in Dent's disease and hypophosphatemic rickets is to normalize serum alkaline phosphatase and achieve longitudinal growth. Conventional treatment with oral phosphate and calcitriol can heal rickets, but it does not always raise serum phosphate concentrations significantly, nor does it always normalize linear growth [12].

Both endogenous and exogenous GH result in an increase in GFR. It is likely that the increased GFR is mediated by IGF-1 [17]. It is of concern that long-term rhGH treatment could produce hyperfiltration with resultant glomerulosclerosis and an accelerated decline in renal function [18]. In our patient's case, GFR, measured as creatinine clearance at the beginning and at the end of monitoring, remained normal, but it increased from 92.8 to 112.7 ml/min/1.73 m^2^. We were also concerned that rhGH might induce hypercalciuria during calcitriol treatment, however calcium excretion did not change significantly.

## Conclusion

Effects of GH therapy in children with Dent's disease and short stature are positive, but it is difficult to reach conclusions because of the small sample size in the literature, the short duration of the therapy and the lack of a control group. Thus, further studies are needed to determinate the pathophysiological mechanism of rhGH action in Dent's disease.

## Abbreviations

GH: gGrowth hormone; GRF: glomerular filtration rate; IGF-1: insulin-like growth factor 1; IGFBP-3: insulin-like growth factor binding protein-3; SD: standard deviation; TRP: tubular reabsorption of phosphate; rhGH: recombinant human growth hormone.

## Consent

Written informed consent was obtained from the patient's mother for publication of this case report and any accompanying images. A copy of the written consent is available for review by the Editor-in-Chief of this journal.

## Competing interests

The authors declare that they have no competing interests.

## Authors' contributions

MS analyzed and interpreted the patient data regarding the endocrinological follow- up and was a major contributor in writing the manuscript. SP and RB performed nephrology management and consulted in the case. ML performed the molecular genetic analysis. All authors read and approved the final manuscript.
